# Ribonucleotide reductase inhibition improves the symptoms of a *Caenorhabditis elegans* model of Alzheimer's disease

**DOI:** 10.1093/g3journal/jkae040

**Published:** 2024-02-27

**Authors:** Ana M Brokate-Llanos, Mireya Sanchez-Ibañez, Mercedes M Pérez-Jiménez, José M Monje-Moreno, Carlos Gómez-Marín, Carlos Caro, Carlos Vivar-Rios, Miguel A Moreno-Mateos, María L García-Martín, Manuel J Muñoz, José L Royo

**Affiliations:** Centro Andaluz de Biologia del Desarrollo, University Pablo de Olavide-CISC-Junta de Andalucía, Ctra Utrera Km 1, Sevilla 41013, Spain; Department of Surgery, Immunology and Biochemistry, School of Medicine, University of Malaga, Boulevar Louis Pasteur s/n, Málaga 29010, Spain; Centro Andaluz de Biologia del Desarrollo, University Pablo de Olavide-CISC-Junta de Andalucía, Ctra Utrera Km 1, Sevilla 41013, Spain; Centro Andaluz de Biologia del Desarrollo, University Pablo de Olavide-CISC-Junta de Andalucía, Ctra Utrera Km 1, Sevilla 41013, Spain; Centro Andaluz de Biologia del Desarrollo, University Pablo de Olavide-CISC-Junta de Andalucía, Ctra Utrera Km 1, Sevilla 41013, Spain; Andalusian Centre for Nanomedicine and Biotechnology (Junta de Andalucía-Universidad de Málaga), BIONAND, Málaga 29590, Spain; Department of Surgery, Immunology and Biochemistry, School of Medicine, University of Malaga, Boulevar Louis Pasteur s/n, Málaga 29010, Spain; Centro Andaluz de Biologia del Desarrollo, University Pablo de Olavide-CISC-Junta de Andalucía, Ctra Utrera Km 1, Sevilla 41013, Spain; Andalusian Centre for Nanomedicine and Biotechnology (Junta de Andalucía-Universidad de Málaga), BIONAND, Málaga 29590, Spain; Centro Andaluz de Biologia del Desarrollo, University Pablo de Olavide-CISC-Junta de Andalucía, Ctra Utrera Km 1, Sevilla 41013, Spain; Department of Surgery, Immunology and Biochemistry, School of Medicine, University of Malaga, Boulevar Louis Pasteur s/n, Málaga 29010, Spain

**Keywords:** Alzheimer, *Caenorhabditis elegans*, RRM2B, gemcitabine

## Abstract

Alzheimer's disease is the main cause of aging-associated dementia, for which there is no effective treatment. In this work, we reanalyze the information of a previous genome wide association study, using a new pipeline design to identify novel potential drugs. With this approach, ribonucleoside-diphosphate reductase gene *(RRM2B)* emerged as a candidate target and its inhibitor, 2′, 2′-difluoro 2′deoxycytidine (gemcitabine), as a potential pharmaceutical drug against Alzheimer's disease. We functionally verified the effect of inhibiting the *RRM2B* homolog, *rnr-2*, in an Alzheimer's model of *Caenorhabditis elegans,* which accumulates human Aβ_1-42_ peptide to an irreversible paralysis. RNA interference against *rnr-2* and also treatment with 200 ng/ml of gemcitabine, showed an improvement of the phenotype. Gemcitabine treatment increased the intracellular ATP level 3.03 times, which may point to its mechanism of action. Gemcitabine has been extensively used in humans for cancer treatment but at higher concentrations. The 200 ng/ml concentration did not exert a significant effect over cell cycle, or affected cell viability when assayed in the microglia N13 cell line. Thus, the inhibitory drug of the RRM2B activity could be of potential use to treat Alzheimer's disease and particularly gemcitabine might be considered as a promising candidate to be repurposed for its treatment.

## Introduction

Alzheimer's disease (Ad) is a progressive and irreversible neurodegenerative disease affecting over 40 million people worldwide (Scheltens *et al.* 2016). Ad is a complex and multifactorial disease produced by a combination of biological, genetic, environmental, and lifestyle factors. Ad is classified between *familial,* or early-onset form, which accounts for less than 1% of the cases and *sporadic* or late-onset Ad , which is the predominant type. Early-onset Ad has been associated with autosomal dominant mutations within the amyloid precursor protein gene and the presenilin 1 and presenilin 2 genes, both encoding for the catalytic subunits of the gamma-secretase intramembrane protease complex (Waring and Rosenberg 2008). On the other hand, late-onset Ad has a polygenic nature, with heritability ranging from 60 to 80% (Wingo *et al.* 2012). The complexity of late-onset Ad relies on the combination of a genetic background with a plethora of environmental factors that finally trigger the disease (Blennow *et al.* 2006). The better-known genetic risk factor in late-onset Ad is the presence of the ε4 allele of the apolipoprotein E gene, although the biochemical networks that affect late-onset Ad comprise a broad spectrum of metabolic pathways (Recabarren and Alarcon 2017).

In the last decades, productivity in pharmaceutical research and development against Ad has decreased dramatically (Raghavendra *et al.* 2012) and only symptomatic treatments that counteract cognitive and behavioral progression are developed (Winblad *et al.* 2016). Cholinesterase inhibitors, for instance, favor interneuronal communication via an increase of acetylcholine levels, which are usually depleted in the damaged central nervous system (CNS). This activity usually improves neuropsychiatric symptoms, such as agitation or depression. On the other hand, memantine works as a low affinity non-competitive antagonist of glutamatergic N-methyl-D-aspartic acid receptors, inhibiting the prolonged Ca^2+^ flow from the extra-synaptic receptors, which is the main cause of neuronal excitotoxicity. These properties contribute to preserve the receptor function at the synapses slowing Ad progression (Xia *et al.* 2010). Additional drugs such as antidepressants are used to help the control of behavioral symptoms. However, current pharmacopeia does not modify, in an effective way, the cognitive decline that accompanies the disease progression (Norton *et al.* 2014; Cappa 2018). The majority of putative disease-modifying treatments under development for Ad target the Aβ peptide, mainly through administration of exogenous monoclonal antibodies. However, this approach has been fraught with failure and confusing results, that differ considerably depending on the Aβ conformations targeted (i.e. monomers, oligomers, protofibrils, fibrils) (van Dyck 2018). Efforts to develop new therapies against Ad suffer from extraordinarily high failure rates that make it difficult to justify continued investment in the field. An analysis of publicly available data from the Phase II studies for Bapineuzumab and Solanezumab indicates that neither compound produced compelling evidence that would justify their progression into further clinical trials (Gold 2017).

Under this scenario, drug repurposing strategies emerge as a feasible solution to overcome the industrial challenges, lowering investment requirements in terms of both time and cost. Drug repurposing is defined as the reuse of known drugs and compounds for a new therapeutic indication (Ashburn and Thor 2004). This strategy has several advantages over the traditional approach to the discovery of new treatments, mainly due to the lower cost of preclinical and clinical phases, and the shorter time for approval by the competent authorities (Mullard 2012; Cummings and Zhong 2014). At present, there are some candidates at different testing levels, such as Rosiglitazone, initially used in Type II Diabetes, the antihypertensive agent Telmisartan, anti-inflammatory drugs such as cromoglycic acid in combination with Ibuprofen, and other drugs such as valproic acid or methylene blue (Appleby and Cummings 2013). However, their effectiveness has yet to be demonstrated in clinical trials.

In this work, we reanalyzed the results from a genome wide analysis (GWAS) initially reported by Hu and coworkers from Pfizer Inc. (Hu *et al.* 2011) and identified the gene ribonucleoside-diphosphate reductase subunit M2 B (*RRM2B)* as a putative target for pharmacological intervention. To functionally validate our strategy, we used a *Caenorhabditis elegans* model of Ad overexpressing human Aβ_1–42_ demonstrating that either RNAi treatment against the *RRM2B* homolog gene (*rnr-2*) or its inhibition by gemcitabine improves the symptoms of the Ad model. We also provide an initial characterization of its biological effect.

## Materials and methods

### GWAS data and expression-quantitative trait loci (eQTL) analysis

Genetic data were obtained from a previous GWAS study comprising 451,101 SNPs performed on a total of 1,034 late-onset Ad cases and 1,186 controls (Hu *et al.* 2011). SNPs with a *P*-value < 0.01 were selected, providing 5,426 candidate SNPs. As a control, the same number of SNPs was randomly extracted. eQTLs data were downloaded from the *online* repository GTEx portal of MIT and Harvard (GTEx Consortium 2013). eQTLs database comprised 249,322 statistically associated SNPs correlated with the expression levels of any of their surrounding genes. We should highlight that GTEx contains common eQTLs, obtained from donor tissues from unselected population. Nevertheless, only CNS-eQTLs comprised by those from: (Amygdala, Anterior Cingulate Cortex BA24, Caudate basal ganglia, Cerebellar Hemisphere, Cerebellum, Cortex, Frontal Cortex, Hippocampus, Hypothalamus, Nucleus accumbens, Putamen, Spinal cord, and Substantia nigra) were extracted from GTEx Analysis v6p eQTL file and further used in our pipeline. Human genome coordinates used for SNP and refseq gene annotation was hg18. Data mining was performed using R programming language R-Studio v1.1.442. Statistics were performed using Sigma Stat 4.0, GraphPad QuickCalcs (https://www.graphpad.com/quickcalcs/), and IBM SPSS v22.0.

### Candidate drug seek

For intergenic SNPs, the closer distance to the target promoter was taken as a positive score. The associated SNPs were classified taking into account the requirement of either an inhibitor or an activator, based on the odds ratio (OR) together with the target gene mRNA levels. A mutant genotype associated to Ad risk (OR > 1) found to be associated to higher target mRNA levels would require an inhibitory pharmacological strategy. Conversely, if the risk allele is associated with lower levels of mRNA, an activator would be required. The candidate drugs were manually checked for existing approval by the competent authority. The presence of a high confident ortholog in *C. elegans* was another requirement for gene selection. For that purpose, OrthoList (Shaye and Greenwald 2011) was used to identify *C. elegans*–human correlations. This was further verified using basic local alignment search tool (BLAST) (Madden 2013), performing BLASTP with the target genes (Rost 1999). Literature search, Vademecum.es, and DGI databases (Griffith *et al.* 2013; Cotto *et al.* 2018) were used to explore gene-drug interactions. Intersection analysis was plotted using the *online* tool Venny version 2.1 (Oliveros 2023). Finally, potential blood-brain barrier permeability was also scored using the online tool admetSAR (Chen *et al.* 2014).

### *C. elegans* functional assays

N2 was used as the wild-type strain and GMC101 (dvls100 [unc-54p::A-beta-1–42::unc-54 3′-UTR + mtl-2p::GFP]), as the Alzheimer disease's model. This strain expresses Aβ_1–42_ in muscle cells leading to animal paralysis. This paralysis occurred more rapidly and more severely when Aβ_1–42_ is produced at 25°C (McColl *et al.* 2012). For paralysis assays, eggs were laid on plates with and without gemcitabine at 16°C for 4 days to develop up to stage L4. Ninety L4 worms (30 worms per 3 plates) were transferred to respective plates and incubated at 25°C for 18–24 h for observation and quantification of paralysis as previously stated (Chen *et al.* 2015), observation and quantification of paralysis were performed in adult animals every 2 or 3 h, we considered paralyzed worms that were not able to move backward a distance equal to their body size paralyzed RNAi feeding was performed as described by Kamath and Ahringer (2003). L4 hermaphrodites were transferred to empty plasmid control pL4440 or *rnr-2* RNAi plates, and the subsequent generation was analyzed. Fertility analysis was performed as described by Perez-Jimenez *et al.* (2021). Briefly, 10 L4 animals, 1 per plate, of each strain were incubated at 16°C, and numbers of eggs were counted every day until animals do not lay more eggs. Adenosine triphosphate (ATP) measurement was performed using Bioluminesce-based ATP determination Kit PRO from Proteonkinase.de according to manufacturer's instructions and a LB960 Luminometer from Berthold Technologies. The worms were grown in NGM medium without bactopectone. Harvest, extraction, and ATP measurement were performed as previously described by Gatsi *et al.* (2014) but using Pierce BCA Protein Assay Kit for sample protein determination. At least, 50 nematodes per condition were used.

### q-PCRs for RNAi knockdown determination

For RNA extraction, 500 L1 were cultured on pL4440 and rrn-2 RNAi plates for 4 days to develop up to stage L4. They were then transferred at 25°C for 16–18 h and collected in a final volume of 50 μl of M9 buffer. They were treated with 350 μl of trizol and frozen at −80°C overnight. RNA extraction was performed using Invitrogen PureLink RNA Mini Kit under manufacturer's instruction. 1μg of RNA was used for cDNA preparation following the protocol of the supplier's Taqman Reverse Transcription Reagents kit (reference: N8080234). q-PCRs were performed using TB Green Premix Ex Taq II (Tli RNAase H Plus) Kit from Takara Bio with 1 μl of one-fifth dilutions of the cDNAs as template. Primer used for *rnr-2* transcript detection was *rnr-2*_Fwd AGGCTTCGTTCGCTGAAAGA and *rnr-2*_Rev TGGCATCAATCCACGCTTCT. *pmp-3* transcript was used for normalization (*pmp-3*_Fwd AAATCAGCGTCCCGACACAT and *pmp-3* ReV CCGGCCAATCATCCTCTTGA).

### Anti-Aβ western blotting

Animals were treated in RNAi and gemcitabine conditions from eggs, as described earlier. Then, 100 animals were manually collected in M9 buffer after 38–40 and 12–14 h at 25°C for RNAi and gemcitabine conditions, respectively. N2 strain was used as negative control. Samples were sonicated with 1 pulse of 3 s at 10% amplitude in Laemmli buffer 2 × (Tris-HCl 100 mM pH 6.8, SDS 4%, Bromophenol blue 0.005%, Glycerol 20%). Protein concentration was calculated with the RC/DC protein assay kit (BioRad) and 12 ug of each condition was loaded in a 12.5% SDS-PAGE with Tris-Tricine (Tris 100 mM, Tricine 100 mM, SDS 0.1%) as running buffer. After electrophoresis, proteins were transferred to a Nitrocellulose membrane and were immunodetected with the anti-Aβ monoclonal 6E10 (Covance) primary antibody at a 1:1,000 dilution and the anti-mouse StarBright 700 secondary antibody at a 1:10,000 dilution (BioRad). Normalization was performed with the total lane protein signal obtained with 2 ug of each experimental condition loaded onto a 12% TGX stain-free gel (BioRad). Three biological replicates were done. *t*-test was used for statistical analysis.

### Cytotoxicity assays

Murine microglia cell line N13 was cultured in Roswell Park Memorial Institute medium supplemented with 2 mM L-glutamine, 10% fetal bovine serum, and 1% penicillin/streptomycin at 37°C in a humidified atmosphere incubator with 5% CO_2_. Five replicas of N13 cells were plated at a density of 1 × 10^4^ cells/well in a 96-well plate at 37°C in 5% CO_2_ atmosphere. After 24 h of culture, media was replaced with fresh one containing gemcitabine (2′-deoxy-2′,2′-difluorocytidine hydrochloride, Merck, Darmstadt, Germany) in concentrations varying from 200 ng/ml to 10 μg/ml. After 24 h, the supernatant of each well was replaced by 200 μl of fresh medium with 3-[4,5-dimethylthiazol-2-yl]-2,5-diphenyl tetrazolium bromide (metabolic activity assay (MTT)) (0.5 mg/ml). After 2 h incubation, the medium was removed, the formazan crystals were solubilized in 200 μl of DMSO, and the solution was vigorously mixed to dissolve the reacted dye. The absorbance of each well was read on a microplate reader (Dynatech MR7000 instruments) at 550 nm.

### Flow cytometry cell cycle analysis

N13 cells were seeded overnight in 12-well plates at a density of 120,000 cells per well, in a final volume of 1 ml. After 24 h, the medium was replaced with fresh one containing gemcitabine at different concentrations. After 24 h, cells were fixed in ice-cold 70% ethanol for 1 h and then incubated with 0.1% RNAse and 50 μg/ml propidium iodide at 37°C for 30 min before flow cytometry sorting and DNA content analysis using CellQuest software.

### Cell morphology studies and live-dead assay

N13 cells were plated at 10^4^ cells/well in a 96-well plate and incubated at 37°C in 5% CO_2_ atmosphere. After 24 h, the medium was replaced with the one containing gemcitabine ranking from 200 ng/ml to 10 µg/ml. After 24 h, Triton X-100 was added to the positive control wells. After 15 min, all the wells were stained with DAPI (4′,6-diamidino-2-phenylindole at 1:3,000) to label all nuclei, although with stronger labeling in living cells, and TO-PRO-3 Iodine (1:1,000) to label only dead cells. Cell morphology images were acquired using a Perkin Elmer Operetta High Content Imaging System with a 20 × LWD 0.45 NA air objective lens. Five well replicas for each condition were analyzed with 10 random image fields captured per well. For each field, fluorescence images for DAPI and TO-PRO-3, as well as a bright-field image were captured. Cell mortality percentages were calculated automatically by Operetta Harmony software, whereby all nuclei (dead and alive) were identified from the DAPI staining and the percentage of dead cells was then determined by the number of nuclei also showing high levels of TO-PRO-3 staining.

## Results

### GWAS data analysis

**Fig. 1.**
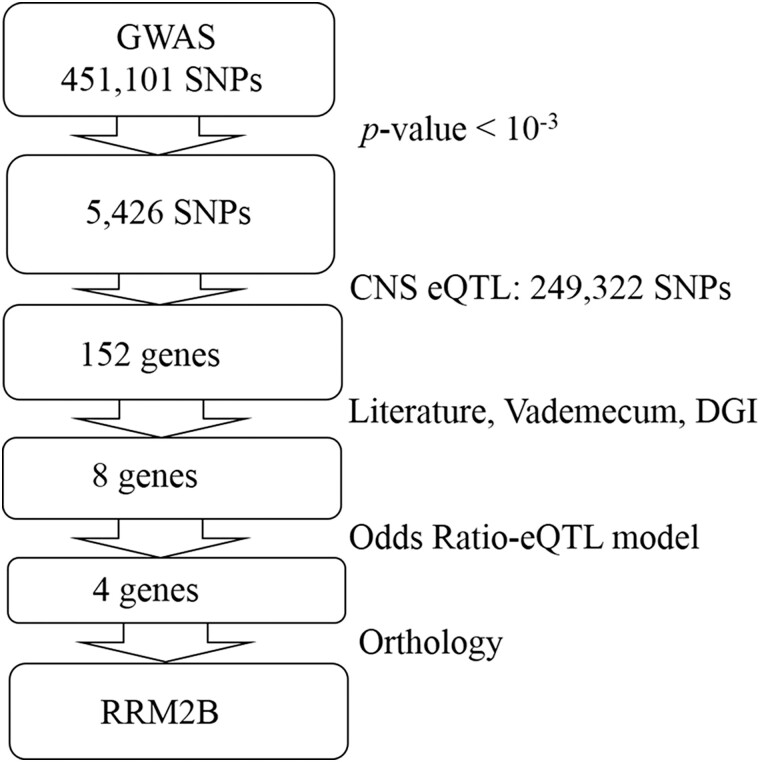
Bioinformatic pipeline followed in the repurposing strategy.

Genetic data from the GWAS study by Hu *et al.* comprised 451,101 SNPs (Hu *et al.* 2011). These were first ranked according to their *P*-value. An initial characterization of the 5,426 top-scoring (χ^2^*P*-value < 10^−3^) associated SNPs did not evidence any enrichment on intragenic variants. These represented 47.71% of the total, approximately the same percentage observed from the 5,426 control random SNPs (49.13%, χ^2^*P*-value = 0.680). Among the statistically significant SNPs, intragenic variants exhibited a slightly higher OR than intergenic SNPs (1.041 ± 0.013 vs. 1.028 ± 0.012; average ± standard deviation), however, these differences were not statistically significant (Student *t*-test *P*-value = 0.130). To date, previous studies have associated a total of 27 genes with different effect sizes on Ad development (Giri *et al.* 2016). The statistically significant intragenic SNPs pointed to 1,346 genes, from which only 6 (0.4%) coincided with any of the previously associated ones. This suggested that many of the associations might be captured by intergenic rather than intragenic variants. Thus, we outcrossed the significant SNP selection with the data available regarding CNS-eQTLs as stated in materials and methods. This approach resulted in an initial set of 152 candidate genes that were at the same time eQTL in the Central Nervous System and associated with Ad with a *P*-value < 10^−3^. This 152 candidate genes list was outcrossed with the information available in the literature, in order to find potential candidate drugs that antagonize the eQTL effect. We observed that only 8 were pharmacologically intervenable with approved drugs. From these, only 4 candidates fitted the drug-target interaction according to the OR-eQTL criteria. Finally, only the RRM2B showed a clear ortholog in *C. elegans* (*rnr-2*), with at least 40% identity and therefore, it was selected for the in vivo assays in this model. See the graphical pipeline in Fig. 1.

### Inhibition of *rnr-2* gene by RNAi or drug treatment reduces paralysis in a *C. elegans*Ad model

**Fig. 2.**
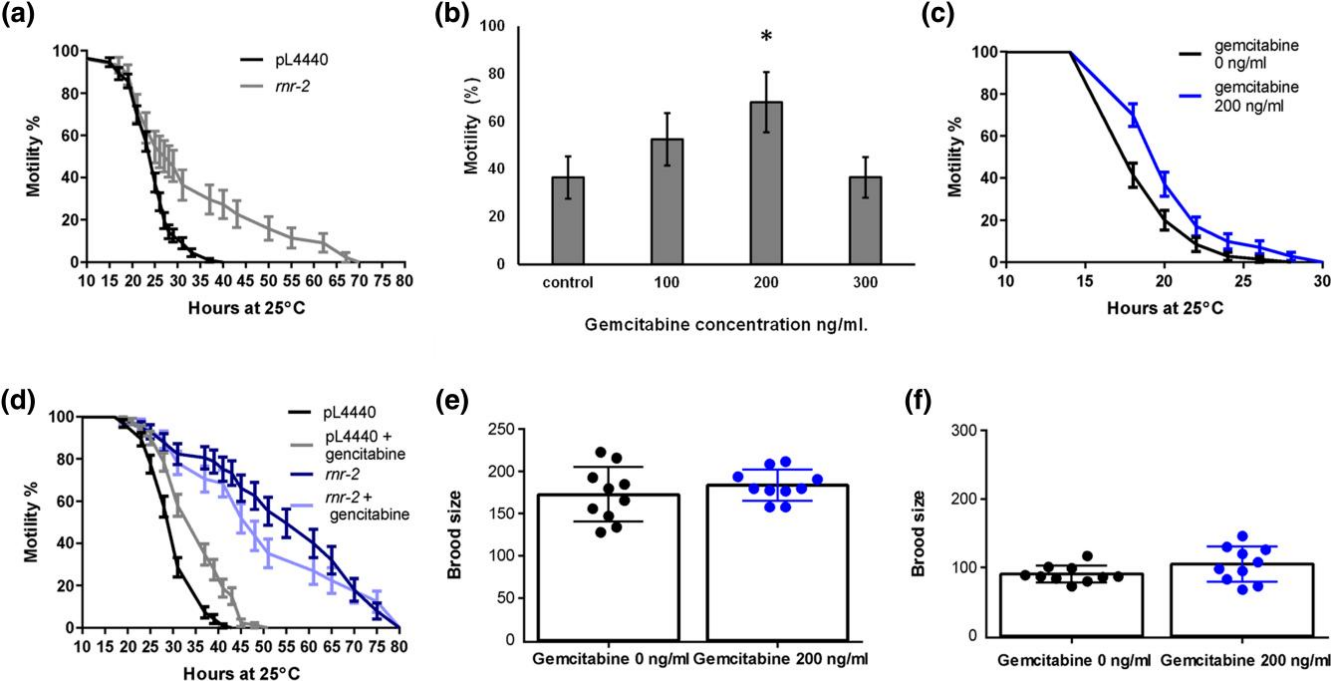
Effects on paralysis rate upon inhibition of *rnr-2* activity in the Ad*C. elegans* model GMC101 strain. a) Motility (percentage of animals non-paralyzed) of GMC101 in *rnr-2* RNAi. Kaplan–Meier representation of the motility shown by GMC101 treated with *rnr-2* RNAi (gray) or with control which consists in the empty plasmid (black). Curves are significantly different *P* < 0.0001. b) Motility rates observed on the GMC101 strain cultured in the presence of different gemcitabine concentrations after 16 h of adulthood at 25°C. Data represented correspond to the average and error bars reflect standard deviation. A minimum of 3 independent experiments were performed for each condition. c) Motility of GMC101 in gemcitabine 200 ng/ml. Kaplan–Meier representation of the motility shown by GMC101 treated with and without gemcitabine 200 ng/ml and incubated at 25°C. Notice that treated nematodes show a delay in paralysis. Curves are significantly different *P* < 0.0001. d) Motility of GMC101 in gemcitabine 200 ng/ml and *rnr-2* RNAi simultaneously (light blue), compared with controls, empty plasmid (black line), gemcitabine (gray line), and *rnr-2* RNAi (dark blue). Double treatment does not further increase the percentage of motile animals. The treatment was done as described earlier. e) Fertility of the wild-type strain treated with 200 ng/ml gemcitabine. The number of descendants per individual was not affected by the treatment, and f) Fertility of the Alzheimer model's strain treated with 200 ng/ml gemcitabine. The number of descendants per individual was not affected by the treatment. See Supplementary Tables 1 and 2 for additional data.

In order to test if the reduction of the activity of the *rnr-2* gene reduces Ad effect in *C. elegans*, we first, inhibited the *rnr-2* by RNAi treatment (Supplementary Fig. 1) and determined the effect in motility of wild-type (N2) and GMC101. This former strain expresses the β-amyloid in muscle cells causing paralysis with age (McColl *et al.* 2012). GMC101 treated with *rnr-2* RNAi delayed the paralysis compared to the control plasmid (Fig. 2a, Supplementary Table 1).

Next, we addressed a pharmacological approach to block the RRN2B *C. elegans* homolog activity in order to determine whether this strategy would mimic the results obtained using RNAi against *rnr-2*. Previously, it has been shown that gemcitabine inhibits RRM2B function, the mammal ortholog of *rnr-2* (Pereira *et al.* 2004; Wang *et al.* 2007), then we study if treatment of gemcitabine could generate the same effect of *rnr-2* RNAi inhibition. Dose-response assays showed that the motility of the GMC101 animals improved significantly when using 200 ng/ml of gemcitabine (Fig. 2b, Supplementary Table 2). Concentrations below 200 ng/ml were not sufficient to observe a significant effect while higher concentrations were probably toxic and translated into a reduction of motility (Supplementary Fig. 2, Supplementary Table 2).

In order to further characterize the drug effect we conducted a time-course analysis. The worms were distributed on plates with and without gemcitabine 200 ng/ml and worms’ motility was evaluated every hour for 7 h after 16 h incubation at 25°C (Fig. 2c, Supplementary Fig. 3, and Supplementary Table 1). We observed a clear improvement associated with the presence of gemcitabine, which significantly delayed the paralysis of the Ad model. This is in agreement with the previous result, using RNAi against *rnr-2* and indicates that reduction of activity of *rnr-2* gene either by RNAi or chemically protects from the negative effect of the expression of β-amyloid.

We have observed that simultaneous treatment of 200 ng/ml gemcitabine in *rnr-2* RNAi knockdown animals does not improve further the mobility in the Ad model (Fig. 2d, Supplementary Fig. 4). This result suggests that the mechanisms by both treatments affect Ad model must be the same and point to RNR-2 as the target of gemcitabine as described in (Pereira *et al*. 2004; Wang *et al.* 2007).

### Gemcitabine treatment does not reduce fertility in *C. elegans*

*rnr-2* gene is expressed in different cells and tissues of the nematode, among them the expression is observed in gonads and germ line (Grun *et al.* 2014; Han *et al.* 2017). The function in these tissues has been considered necessary for correct development of gonad and fertility and consequently, reduction of activity reduces fertility (Green *et al.* 2011). As described before, in our experiments, we also observed a reduction of fertility in animals treated with *rnr-2* RNAi, this phenotype could be generated by germline reduction or by other mechanisms. Depletion of germ line, but not other cells of the gonad, generates a protective signal that reduces proteotoxicity (Wei and Kenyon 2016). In order to rule out if the protective effect of the gemcitabine treatment is due to a reduction of germ line, we treated wild-type nematode with 200 ng/ml of gemcitabine and found that there is no reduction of fertility compared to non-treated nematodes (Fig. 2e, Supplementary Fig. 5). Similar result was observed when treated the strain used as the Alzheimer's model (GMC101) (Fig. 2f). These results indicate that the protective effect of gemcitabine is not due to a reduction of germ line.

### Gemcitabine treatment does not reduce β-amyloid aggregates

**Fig. 3.**
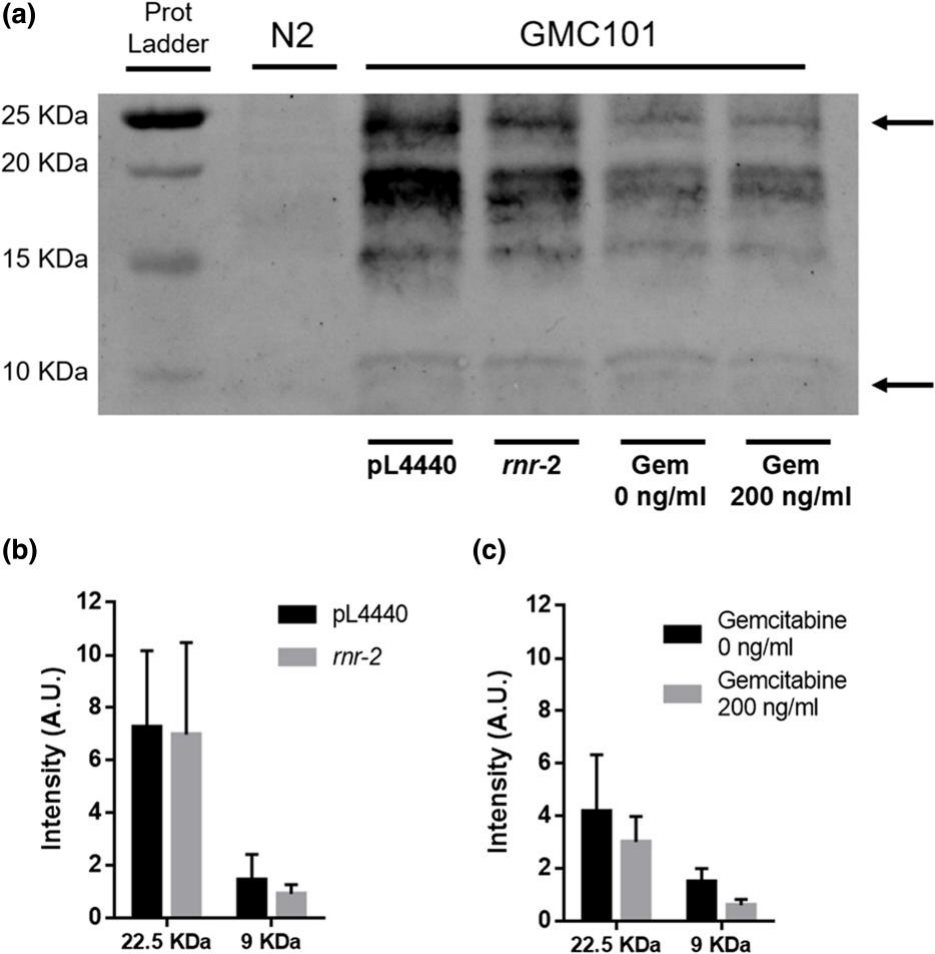
Western blot analysis of *rnr-2* RNAi and gemcitabine treatment. a) A representative western blot of β-amyloid species present in the Alzheimer's model GMC101 strain. N2 is the wild-type strain where no signal is detected. (pL4440) Animals feed on HT115*E. coli* strain with empty vector. (*rnr-2*), animals feed on HT115*E. coli* strain with *rnr-2* RNAi (gem 0 ng/ml), animals feed on OP50*E. coli* strain without treatment (gem 200 ng/ml), and animals feed on OP50*E. coli* strain with 200 ng/ml of gemcitabine. Food source seems to affect general levels of β-amyloid. See full-length gel in Supplementary Fig. 7. b) Quantification of the 22.5 kDa band (large oligomers) and 9 kDa (small oligomers) in a *rnr-2* RNAi treatment or control pL4440. No significant different is observed for any of the oligomers sizes in any of the treatments. Three biological replicates for each sample were done using *t*-test analysis, and c) Quantification of the 22.5 kDa band (large oligomers) and 9 kDa (small oligomers) in animals is non-treated or treated with 200 ng/ml of gemcitabine. No significant different was observed for any of the oligomers sizes in any of the treatments. Three biological replicates for each sample were done using *t*-test analysis.

To determine whether the reduction of activity of *rnr-2* generates changes in the β-amyloid aggregates composition, we performed a western blot analysis to identify changes in the size of the aggregates under the different treatments. *rnr-2* RNAi treatment and its empty plasmid control (pL4440) are done with the nematodes feeding on HT115*Escherichia coli.* The gemcitabine treatment and its control are done using the OP50*E. coli* strain. This difference in food source and other experimental conditions seem to affect general levels of β-amyloid. When comparing larger oligomer of β-amyloid (22.5 kDa) between *rnr-2* RNAi and its control (pL4440) we did not observe a significant reduction of those aggregates neither significant changes in the smaller aggregates (9 kDa). Similar results are observed when comparing animals treated with gemcitabine with non-treated nematodes. In this case, we can observe a slight reduction in both large and small aggregates but no statistically significant (Fig. 3). These results indicate that the beneficial effect of the treatment is not due to the reduction of aggregates but more probably to a resistance to the toxicity of the *β*-amyloid aggregates.

### Gemcitabine treatment increases ATP cell content

**Fig. 4.**
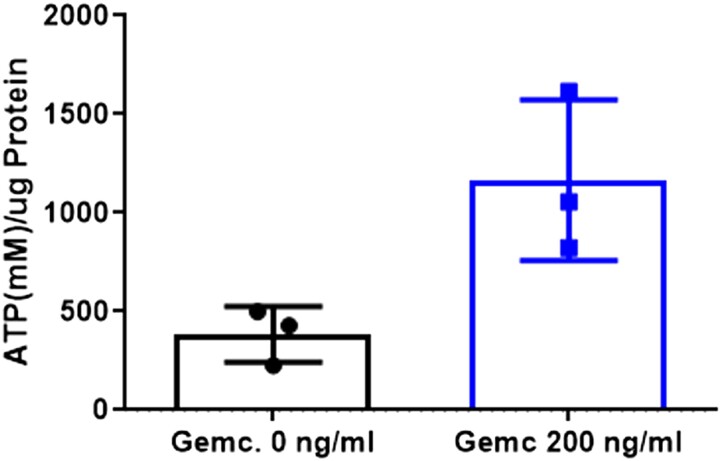
Effect of gemcitabine treatment in ATP level in wild type. Average and standard deviation are represented. Measurements were performed 18 h after incubation at 25°C, with minimum of 50 animals per condition (*N* = 3). ATP level is higher in the wild-type strain (N2). U-Mann–Whitney test, *P-*value = 0.0349.

At this point, we wondered whether the effect of gemcitabine could be linked to a greater resistance to the Aβ plaques induced toxicity. Different authors found that nucleotides, specifically ATP, accumulate after exposure to gemcitabine (van Moorsel *et al.* 2000; Guo *et al.* 2016). This increase in the bioavailable amount of ATP could allow the cells to combat the cytotoxicity produced by the accumulation of the toxic peptide. To explore whether this could be the mechanism by which animals overexpressing Aβ_1–42_ delayed their paralysis, we measured intracellular ATP from N2 and GMC101 strains, with and without gemcitabine, at 25°C. Treatment with 200 ng/ml of gemcitabine increased the amount of ATP 3.03 times in the N2 wild-type strain (384.9 nM ATP/ug of protein) vs. non-treated (1,165 nM ATP/ug of protein; *t*-test, *P*-value = 0.0349) (Fig. 4, Supplementary Fig. 6). Similar results are observed in the strain GMC101, where the gemcitabine treatment also increased the ATP levels (Supplementary Fig. 6).

### Protective dose for *C. elegans* is not toxic in N13 cell line

**Fig. 5.**
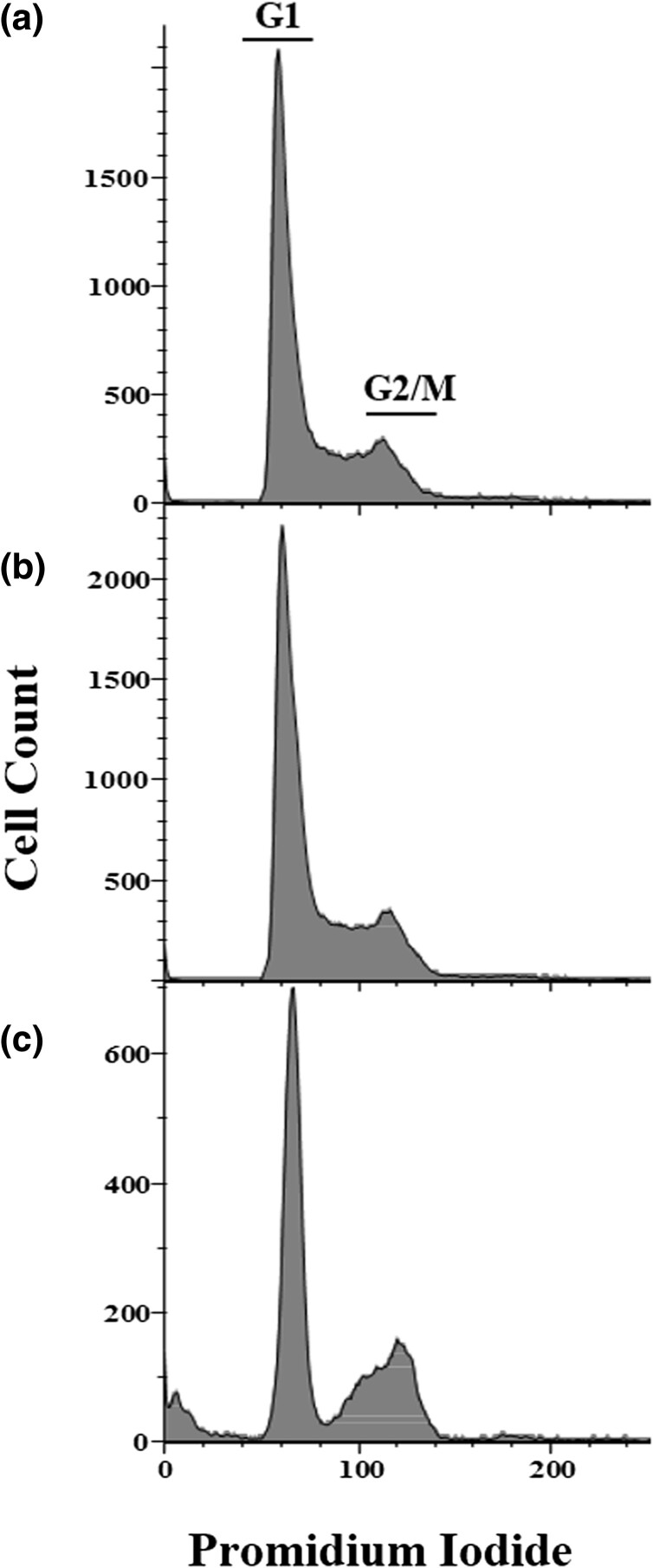
Cell cycle analysis using flow cytometry N13 cells exposed to different gemcitabine concentrations. Histograms represent the cell count according to their DNA content, measured with propidium iodide. Cell cycle phases G1 and G2/Mitosis are highlighted on the upper panel and can be interpolated to the medium and lower panels. Interphase (S phase) cell proportion was calculated as those between G1 and G2/M. Panels represent the following experimental conditions: a) Untreated culture. b) Presence of gemcitabine 200 ng/ml, and C) Presence of gemcitabine 3 μg/ml.

Typical gemcitabine effective doses for cancer treatment range between 3 and 42 μg/ml. These treatments often resulted in bone marrow dysfunction and very common side effects including shortness of breath, low white and red blood cell count and low platelet count, vomiting and nausea, elevated transaminases, skin rashes and itching, hair loss, and edema. Therefore, a fundamental observation from our data is that the efficient concentration needed in the Ad*C. elegans* model is 10–20 times lower than that used in chemotherapy. In order to characterize the cytotoxicity of gemcitabine 200 ng/ml, we performed different assays. First, the murine N13 glial cell line was cultured and exposed to different gemcitabine doses and its effect on the cell cycle was analyzed by flow cytometry. The proportion of cells that remained in S phase of the cell cycle was virtually identical in the negative control (17%, *n* = 6,367) and the sample at 200 ng/ml gemcitabine (18%, *n* = 7,901) (χ^2^*P*-value = 0.122). However, a dose of 3 μg/ml significantly altered the cell cycle, preventing the cells from progressing from G1 to S phase resulting in a decrease in the proportion of interphase cells (12% *n* = 1,499) (χ^2^*P*-value < 10^−3^) (Fig. 5).

**Fig. 6.**
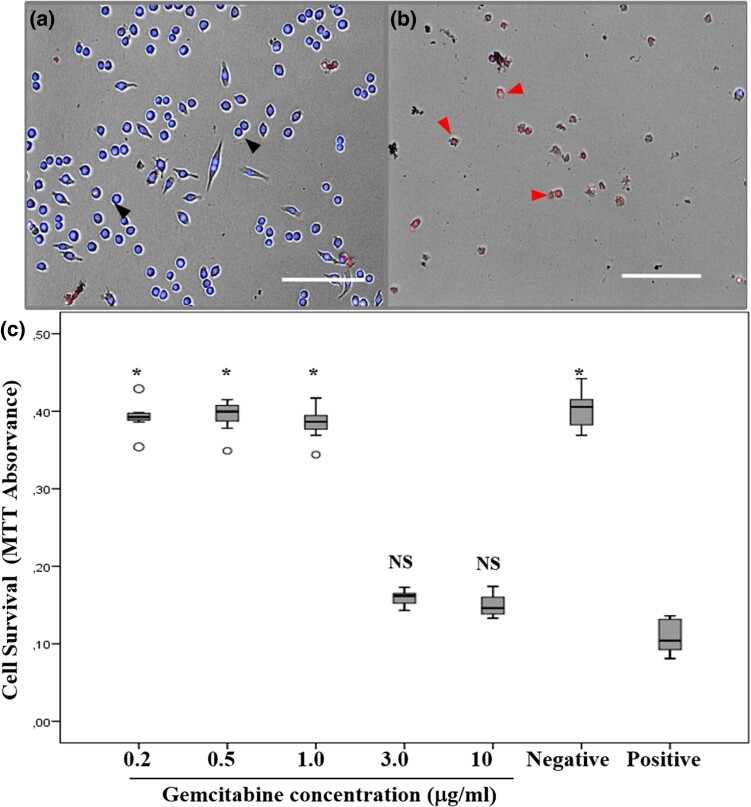
Survival assays of N13 cells exposed to different gemcitabine concentrations. The images show the merge of DAPI (blue) and TO-PRO-3 iodine (red). a) Cell exposed to gemcitabine concentration of 0.2 μg/ml. b) Cell exposed to gemcitabine concentration of 3 μg/ml. Black arrows show healthy cells and red arrows the apoptotic cones (scale bar corresponds to 100 μm), and c) MTT viability assay using different gemcitabine concentrations on N13 cells. Box plots represent the mean and quartiles 1 and 4. Error bars represent the range. Asterisks represent *P* < 0.05 in the Kruskal–Wallis non-parametric test taking the positive control as a reference. Not statistically significant results are highlighted as “NS.”

Next, we evaluated cytotoxicity using a colorimetric cell MTT. Upon exposure of cells to 1 μg/ml of gemcitabine, no effect over mitochondrial activity was detected, whereas exposure to 3 μg/ml decreased their mitochondrial activity below 40%, as compared to its negative control (Fig. 6a). Nevertheless, to ensure a proper evaluation of the gemcitabine cytotoxicity over N13 cells, a “live-dead” assay and the evaluation of cell morphology were also performed. As mentioned in *Materials and methods* section, TO-PRO3 dye is able to label dead cells, while DAPI dye labels living cells. Thus, cells exposed to 3 µg/ml were labeled by TO-PRO3 dye in a percentage close to 100%, whereas the majority of cells exposed to 0.2 µg/ml were labeled by DAPI. In addition, cells exposed to 3 μg/ml changed their morphology, suffering cell shrinkage and fragmentation into membrane-bound apoptotic bodies (Fig. 6b) On the other hand, cells exposed to 0.2 μg/ml did not show any of these changes (Fig. 6c). Furthermore, the live-dead assay showed a significant decrease in the total number of cells (per field), from more than 2,000 cells in the controls to less than 250 cells in those exposed to a concentration of 3–10 μg/ml gemcitabine. In contrast, in the rest of evaluated concentrations, the total number of cells per image remained similar to the untreated control. Moreover, not only a decrease in the total number of cell was observed in these wells, but it was also observed that the cells that were still attached to the bottom were almost dead, since nearly 80% were labeled with TO-PRO3.

## Discussion

Here, we describe a drug repurposing study that has been carried out using the GWAS results from a previous work reported by Hu *et al.* which involved > 450,000 genetic markers randomly distributed throughout the genome. These markers were genotyped in 1,034 Alzheimer's cases and 1,186 controls. Our re-analysis began analyzing the 5,426 genetic signals with the highest statistical significance. These data were intersected with the ones correlating SNPs with gene expression in the CNS to determine which of our genetic signals were linked to differential expression levels. Thus we obtained a list of *loci* epidemiologically linked to Alzheimer's disease and with evidence of a functional correlate on gene expression. Using different drug databases, potential target drugs known to be modifiers of our genes of interest were postulated and hierarchized. The best of our candidates was the *RNR2B* gene, whose antagonist is gemcitabine. Gemcitabine is a nucleoside analog drug, approved by the United States Food and Drug Administration in 1996 and commonly used as a treatment for solid tumors, administered intravenously and usually used in conjunction with other drugs to increase its cytotoxic effect (Fischer *et al.* 2010). Under this scenario, we proceeded to its functional characterization. The experiments were performed on a *C. elegans* strain expressing the human form of Aβ_1–42_ in muscle cells, so that the tissue damage is evidenced in the form of paralysis of the animals. Our data revealed a significant increase in motility when concentrations of 200 ng/ml of gemcitabine were used. The inhibition of the target gene by means of RNAi reflected the same trend and simultaneous treatment does not generate a further improvement, suggesting that gemcitabine acts through inhibition of RNR-2 to generate the beneficial effect. Other treatments that have a beneficial effect on *C. el*egans model of Alzheimer's disease, like the natural compounds *Gingko biloba* extract, reduce Aβ oligomer formation and as a consequence the monomers observed increase (Wu *et al.* 2006). We do not observe reduction of Aβ oligomer formation under the treatments and neither increase of monomers which indicates that the improvement of the symptoms in the Alzheimer's model is not due to reduction of Aβ oligomer formation and most probably to increase of resistance to its toxicity. The overproduction and accumulation of Aβ are keys for the progression and pathogenesis of Alzheimer's disease. Due to the oxidative stress produced by senile plaques, the ribonucleotide reductase enzyme, which is involved in the regulation of inflammatory pathways (Jones and Kounatidis 2017; Kheiri *et al.* 2018), mitochondrial homeostasis (Diaz 2010; Lunnon *et al.* 2017; Shoshan-Barmatz *et al.* 2018), and cell cycle arrest (Nagy 2007), is activated. A possible pathogenic pathway to delve into the positive effect of gemcitabine is mitochondrial dysfunction. Increasing evidence indicates that the accumulation of Aβ inhibits mitochondrial enzymes such as cytochrome-c oxidase, resulting in faults such as increased production of free radicals or low ATP production (Onyango *et al.* 2016). Based on this pathological basis, we could postulate that a partial block of the RRM2B activity would increase the intracellular concentrations of ATP as described previously (van Moorsel *et al.* 2000; Guo *et al.* 2016), compensating the cytotoxicity produced by the accumulation of Aβ and therefore improving muscle mobility problems. When these experiments were performed in the *C. elegans* model, we found that the inhibition of RRM2B using gemcitabine 200 ng/ml significantly increases intracellular ATP levels, which might be the biological basis of the observed improvement. Other authors have related increased ATP levels with improvement of the symptoms of Ad in *C. elegans* model (Griñán-Ferré *et al.* 2021; Liang *et al.* 2021), as also in humans where, intravenous ATP has been approved for a clinical trial to improve Ad progression (Varea 2017).

From a clinical perspective, we should highlight that the proposed gemcitabine concentration in *C. elegans* is approximately 10–20 times lower than used for chemotherapy, although the effective concentration in patient should be determined. This might be of importance when facing a potential clinical use. For this reason, we conducted a characterization of the potential cytotoxicity of the proposed gemcitabine dose. Cell cycle data, as well as the in vitro toxicity data in cell cultures, reveal 200 ng/ml as practically harmless. As consequence, we propose gemcitabine shall be considered for further evaluation as a potential therapeutic agent against in Alzheimer's disease.

## Supplementary Material

jkae040_Supplementary_Data

## Data Availability

Strains and plasmids are available upon request. The authors affirm that all data necessary for confirming the conclusions of the article are present within the article, figures, and tables. Supplemental material available at G3 online.
