# Supplementary material for: Ribonucleotide reductase inhibition improves the symptoms of a Caenorhabditis elegans model of Alzheimer's disease
Source: G3 (Bethesda). 2024 Feb 27;14(5):jkae040. doi: 10.1093/g3journal/jkae040 (PMC11075554; doi:10.1093/g3journal/jkae040)
Supplement: jkae040_Supplementary_Data [file jkae040_supplementary_data.zip › Supplementary_Table_1_G3-2023-404727.docx]

**Supplementary Table 1:** Data of the motility curves under inhibition of *rnr-2* gene by either RNAi or Gemcitabine treatment.

| **Conditions** | **Paralysis/**  **censored** | **Mean**  **motility (hours)** | **% of**  **control** | ***p* *vs.***  **control** | **Plotted In** |
| --- | --- | --- | --- | --- | --- |
| Control | 112/0 | 23,1 |  |  | 2A |
| *rnr-2* RNAi | 44/0 | 27,3 | 18,18 | <0,0001 | 2A |
|  | | | | | |
| Gemcitabine 0 ng/ml | 70/0 | 16,7 |  |  | 2C |
| Gemcitabine 200 ng/ml | 70/0 | 19,1 | +14,37 | 0,0020 | 2C |
|  |  |  |  |  |  |
| Control | 94/7 | 28,4 |  |  | 2D |
| Control + Gemcitabine 200 ng/ml | 85/17 | 33,3 | +17,25 | <0,0001 | 2D |
| *rnr-2* RNAi | 53/6 | 54,6 | +92,25 | <0,0001 | 2D |
| *rnr-2* RNAi + Gemcitabine 200 ng/ml | 48/14 | 45,5 | +60,21 | <0,0001 | 2D |
|  |  |  | -16,6 *vs* *rnr2* | 0,4244 |  |
|  |  |  |  |  |  |
| Gemcitabine 0 ng/ml | 99/1 | 12,8 |  |  | S3A |
| Gemcitabine 200 ng/ml | 90/2 | 14 | +9,37 | <0,0001 | S3A |
|  |  |  |  |  |  |
| Gemcitabine 0 ng/ml | 94/5 | 16,8 |  |  | S3B |
| Gemcitabine 200 ng/ml | 102/8 | 19,9 | +18,45 | <0,0001 | S3B |
|  |  |  |  |  |  |
| Gemcitabine 0 ng/ml | 81/1 | 16,7 |  |  | S2C |
| Gemcitabine 200 ng/ml | 84/5 | 18,3 | +9,58 | 0,0059 | S2C |
|  |  |  |  |  |  |
| Control | 98/3 | 32,9 |  |  | S4 |
| Control + Gemcitabine 200 ng/ml | 101/6 | 35,7 | +8,51 | 0,0422 | S4 |
| *rnr-2* RNAi | 96/5 | 41,5 | +26,13 | <0,0001 | S4 |
| *rnr-2* RNAi + Gemcitabine 200 ng/ml | 89/5 | 43,6 | +32,52 | <0,0001 | S4 |
|  |  |  | +5,06 *vs* *rnr-2* | 0,6460 |  |
|  |  |  |  |  |  |
