# Supplementary material for: Ribonucleotide reductase inhibition improves the symptoms of a Caenorhabditis elegans model of Alzheimer's disease
Source: G3 (Bethesda). 2024 Feb 27;14(5):jkae040. doi: 10.1093/g3journal/jkae040 (PMC11075554; doi:10.1093/g3journal/jkae040)
Supplement: jkae040_Supplementary_Data [file jkae040_supplementary_data.zip › Supplementary_Table_2_G3-2023-404727.docx]

**Supplementary Table** **2:** Data of motility at different concentration of Gemcitabine

| **Concentrations**  **Gemcitabine ng/ml** | **Motility %** | **SD** | ***p* *vs.* control** | **Plotted In** |
| --- | --- | --- | --- | --- |
| 0 | 36,5 | 8,93 |  | 2B |
| 100 | 52,6 | 10,97 | 0,094 | 2B |
| 200 | 68,16 | 12,6 | <0,0001 | 2B |
| 300 | 36,58 | 8,58 | 0,420 | 2B |
|  |  |  |  |  |
| 0 | 31,93 | 3,36 |  | S2A |
| 300 | 44,79 | 6,55 | 0,029 | S2A |
| 3.000 | 33,36 | 1,93 | 0,798 | S2A |
| 30.000 | 36,26 | 1,97 | 0,452 | S2A |
|  |  |  |  |  |
| 0 | 15,47 | 2,87 |  | S2B |
| 100 | 26,24 | 7,49 | 0,030 | S2B |
| 200 | 31,4 | 1,67 | 0.002 | S2B |
| 300 | 22, | 6,66 | 0.147 |  |
|  |  |  |  |  |
| 0 | 19,28 | 8,63 |  | S2C |
| 200 | 29,86 | 1,93 | 0,008 | S2C |
