# Supplementary figures and images for: Ribonucleotide reductase inhibition improves the symptoms of a Caenorhabditis elegans model of Alzheimer's disease
Source: G3 (Bethesda). 2024 Feb 27;14(5):jkae040. doi: 10.1093/g3journal/jkae040 (PMC11075554; doi:10.1093/g3journal/jkae040)

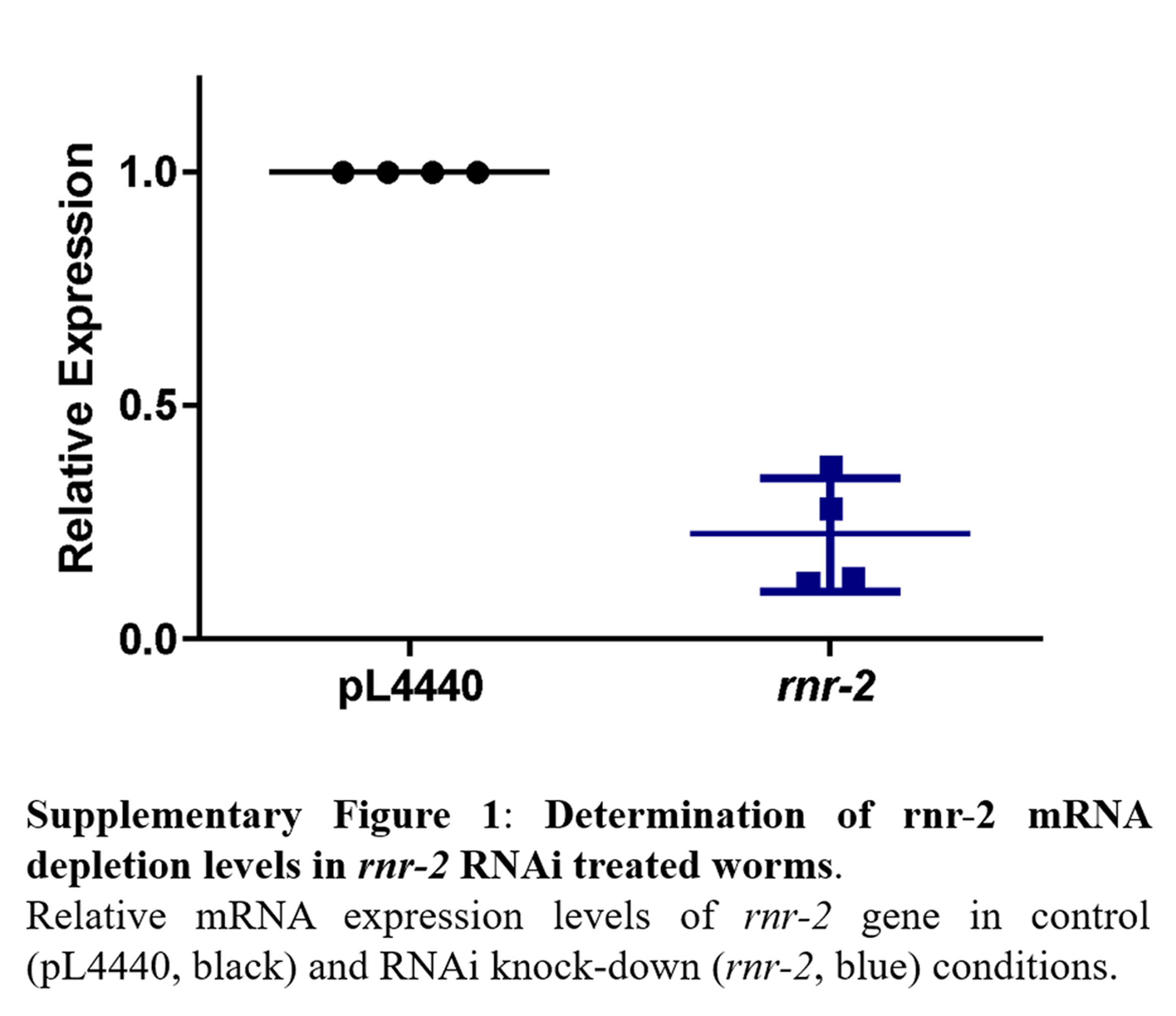

Supplement: jkae040_Supplementary_Data [file jkae040_supplementary_data.zip › Supplementary_Figure_1_G3-2023-404727.tif]

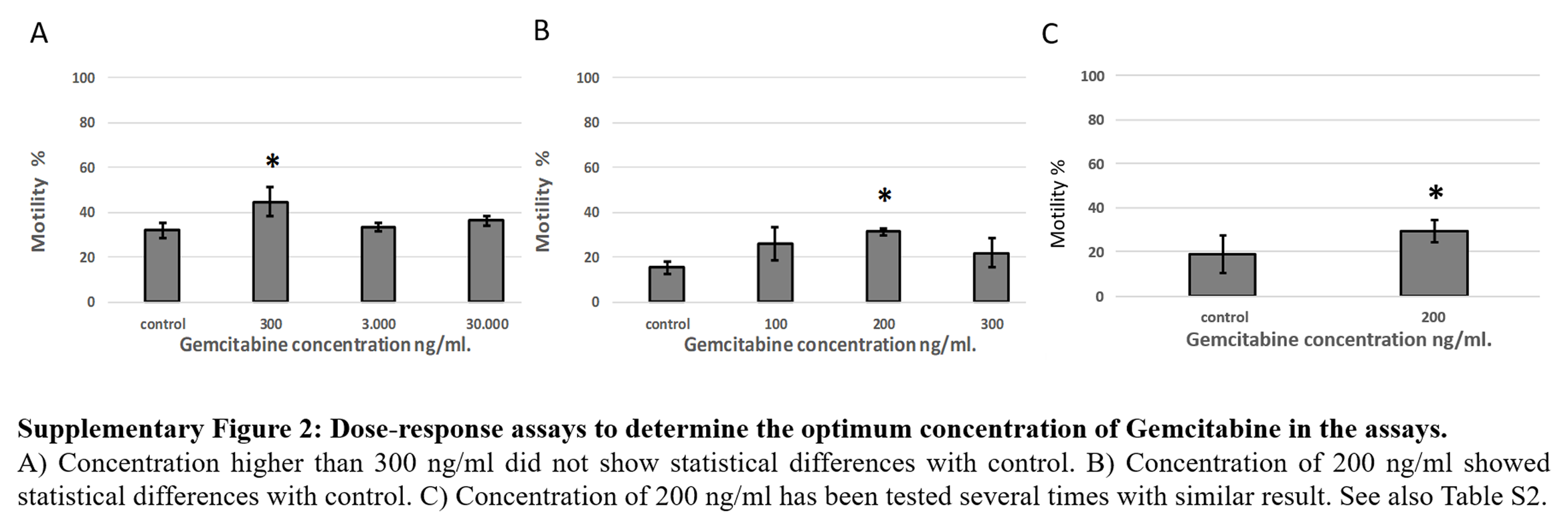

Supplement: jkae040_Supplementary_Data [file jkae040_supplementary_data.zip › Supplementary_Figure_2_G3-2023-404727.tif]

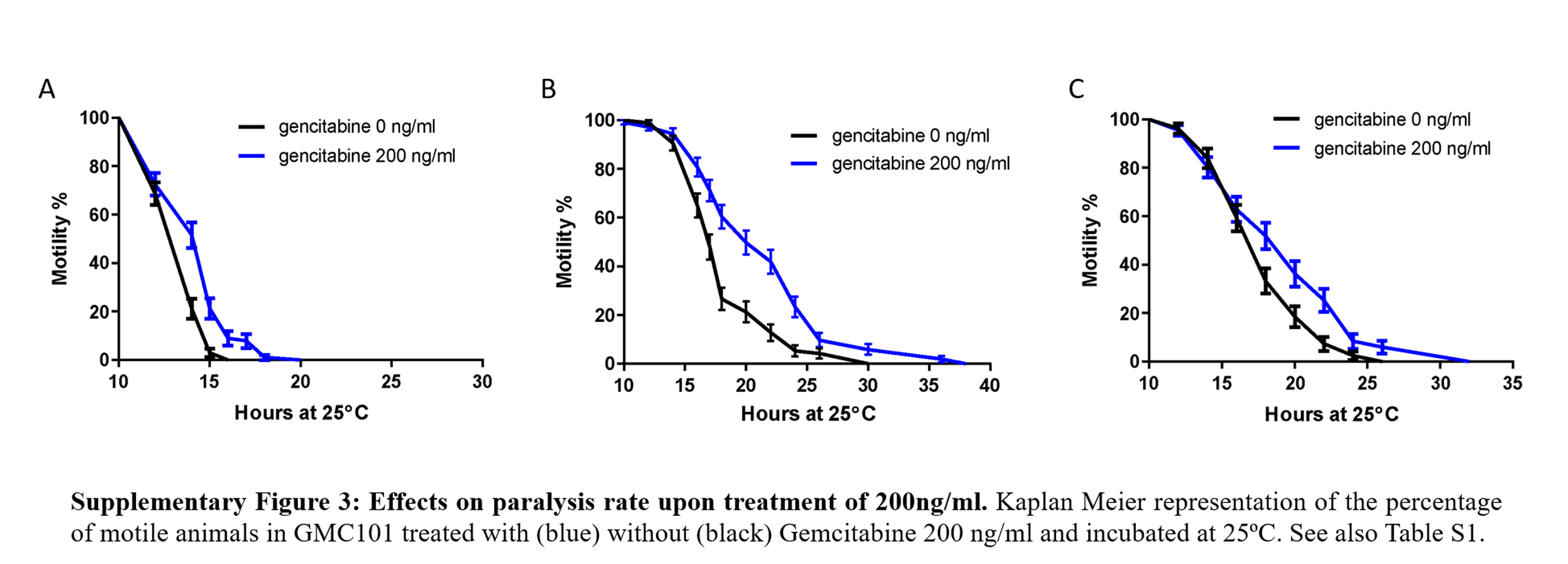

Supplement: jkae040_Supplementary_Data [file jkae040_supplementary_data.zip › Supplementary_Figure_3_G3-2023-404727.tif]

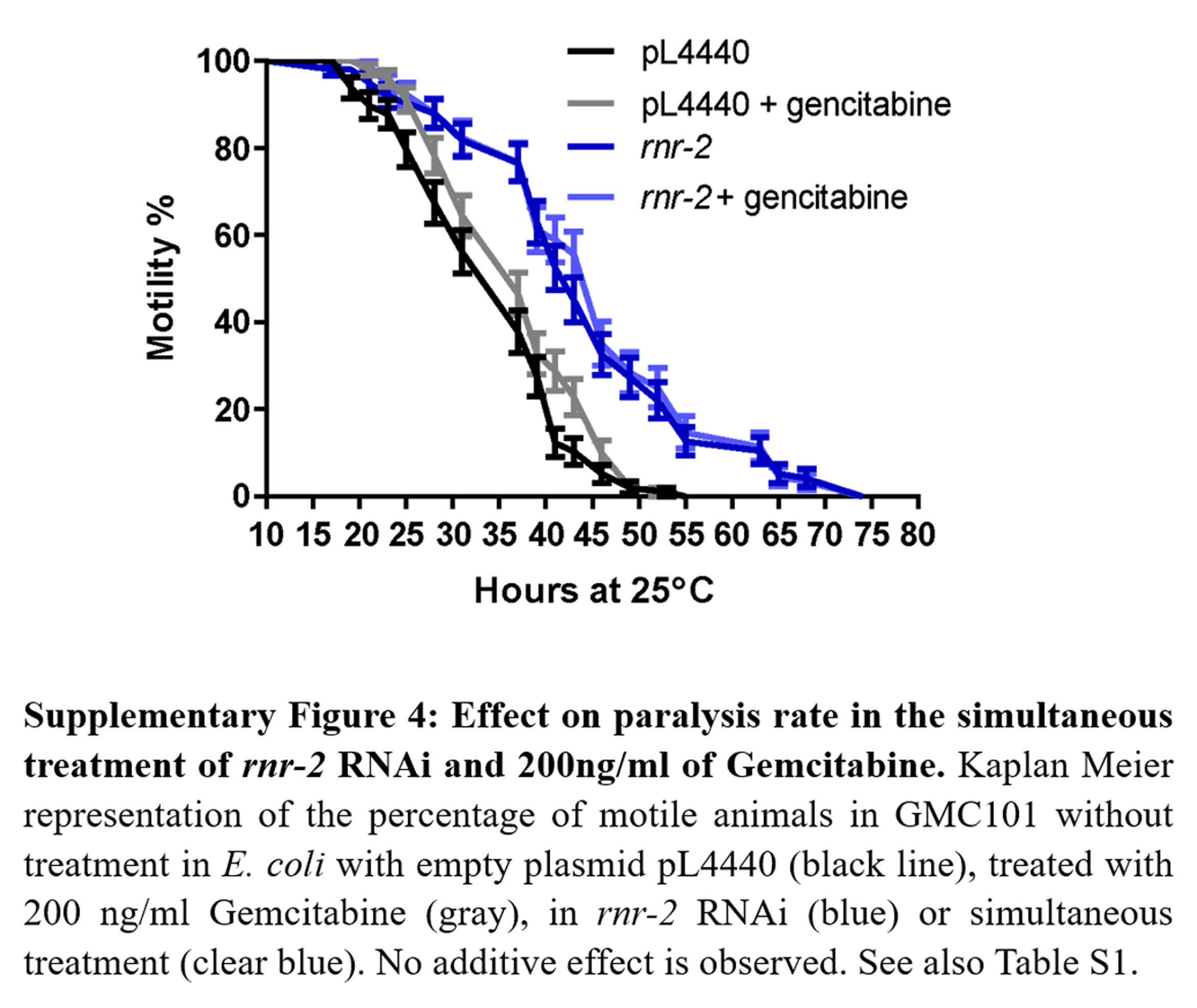

Supplement: jkae040_Supplementary_Data [file jkae040_supplementary_data.zip › Supplementary_Figure_4_G3-2023-404727.tif]

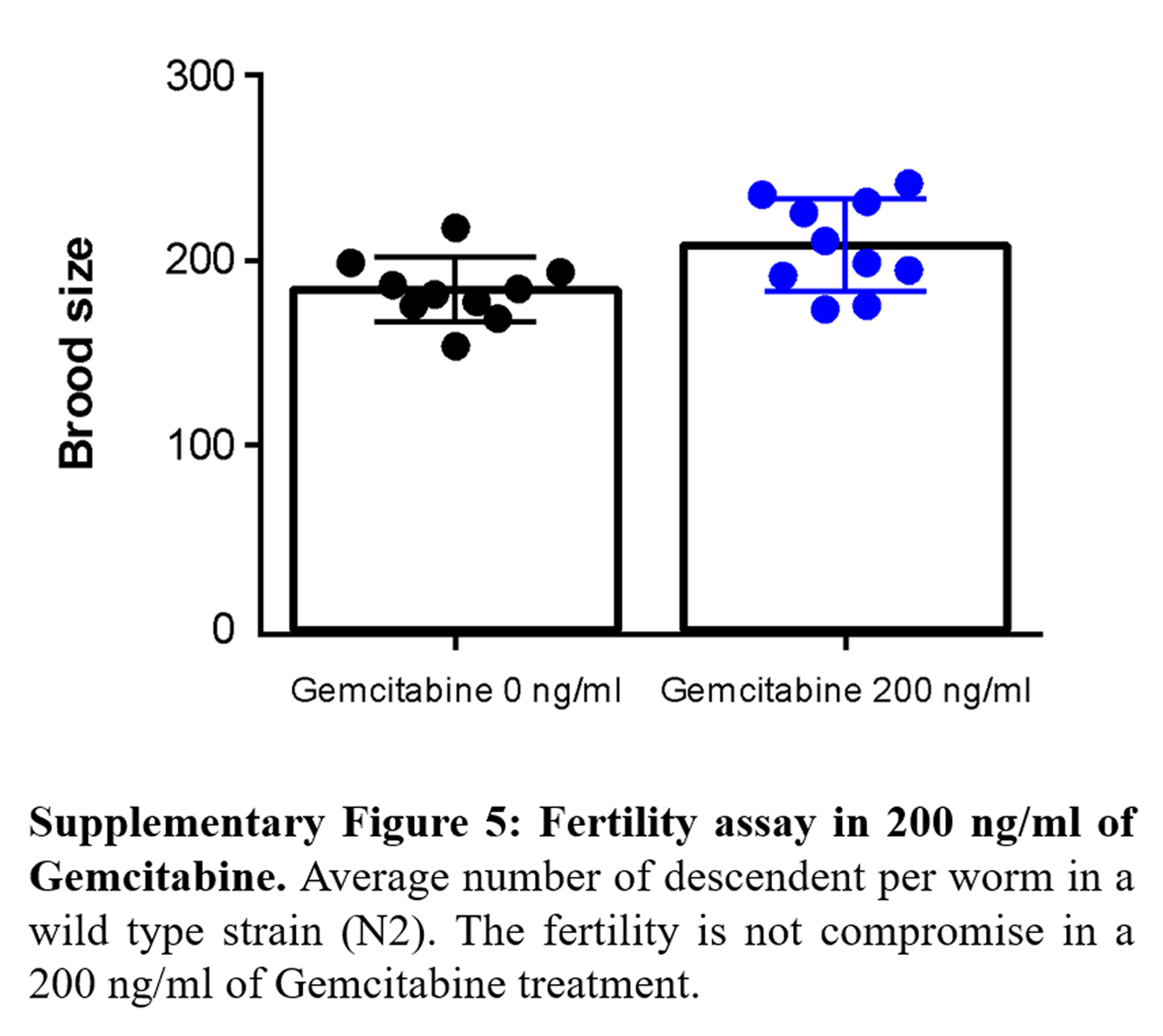

Supplement: jkae040_Supplementary_Data [file jkae040_supplementary_data.zip › Supplementary_Figure_5_G3-2023-404727.tif]

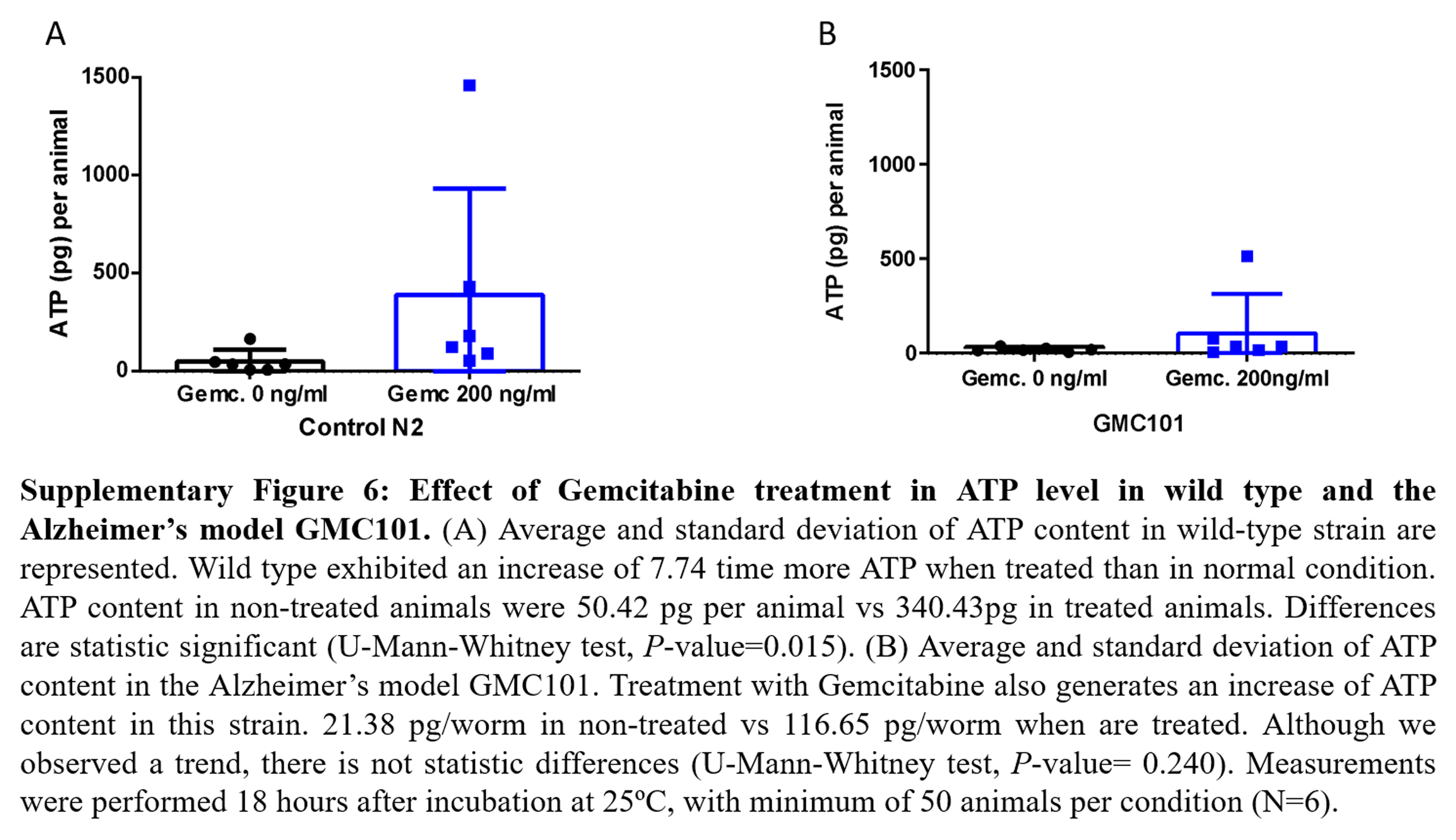

Supplement: jkae040_Supplementary_Data [file jkae040_supplementary_data.zip › Supplementary_Figure_6_G3-2023-404727.tif]

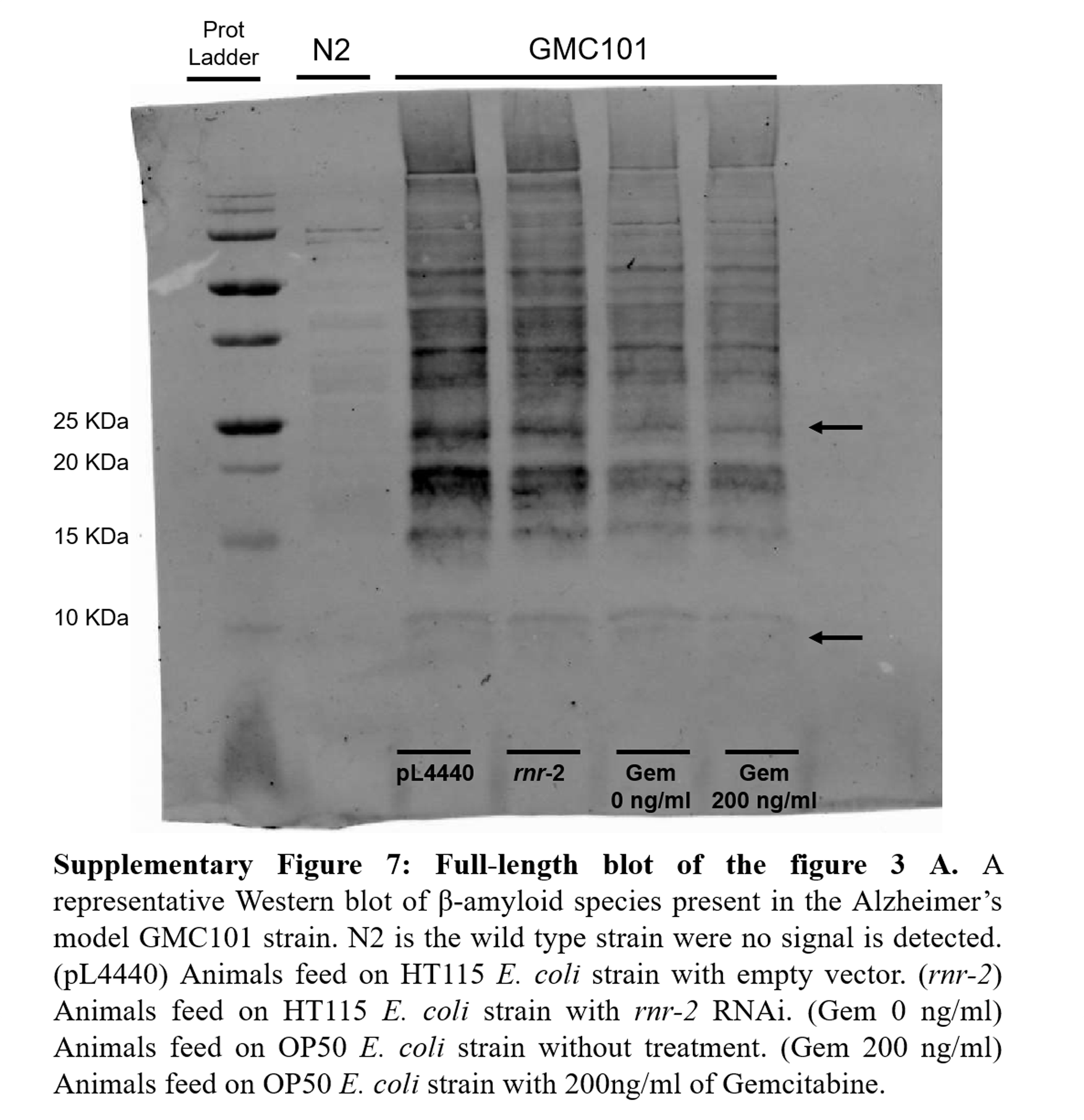

Supplement: jkae040_Supplementary_Data [file jkae040_supplementary_data.zip › Supplementary_Figure_7_G3-2023-404727.tif]
